# The effects of a short-term physician training on smoking cessation in a university pulmonary department

**DOI:** 10.3205/000282

**Published:** 2020-07-03

**Authors:** Anna Bauer, Lorena Brenner, Julia Moser, Franziska Trudzinski, Volker Köllner, Robert Bals

**Affiliations:** 1 Department of Internal Medicine V – Pulmonology, Allergology and Critical Care Medicine, Saarland University, Homburg, Germany; 2 Department of Psychosomatic Medicine, Rehabilitation Center Seehof, Federal German Pension Agency, Teltow, Germany; 3 Psychosomatic Rehabilitation Research Group, Department of Psychosomatic Medicine, Center for Internal Medicine and Dermatology, Charité – Universitätsmedizin Berlin, Germany

**Keywords:** smoking, smoking cessation, counseling

## Abstract

**Objective:** The objective was to evaluate the effect of a short physician training in smoking cessation on the physicians’ performance of smoking cessation interventions. The effects on patients’ cessation rates were analyzed as well. A further aim was to identify barriers for providing cessation interventions. The study was conducted in an acute care pulmonology department of a German university hospital.

**Methods:** 24 physicians of the pulmonology department of a German university hospital received a two-hour training in smoking cessation. 109 pre- and 89 post-training group patients were compared with regard to the frequencies of received smoking cessation interventions (Ask, Advise, Assist) and three- and six-month abstinence rates. Physicians estimated their intervention frequencies and gave reasons for not providing cessation interventions.

**Results:** In a multivariable analysis (p<0.05), the physicians’ application of “Ask” (OR 3.28, 95% CI 1.13–9.53) and the six-month abstinence rates (OR 2.70, 95% CI 1.24–5.84) were significantly higher in the post-training group. The univariate analysis also showed a significant effect on “Assist” (OR 2.05, 95% CI 1.09–3.87). No significant effect was seen on “Advise to quit”. Physicians overestimated their intervention frequencies and reported the patients’ low motivation to stop, an oncological disease and palliative care situation as barriers to performing smoking cessation.

**Conclusion:** A short physician training in a hospital department of pulmonology increases the use of guideline-based cessation strategies and may improve cessation rates. The findings show that hospital-based strategies such as physician trainings could be useful in the improvement of smoking cessation. Strategies for overcoming barriers for providing smoking cessation interventions are needed.

## Zusammenfassung

**Ziel:** Das Ziel war die Evaluation der Effekte einer Ärzte-Kurzschulung zur Raucherentwöhnung auf die Anwendungshäufigkeit der Entwöhnungsstrategien durch die Ärzte. Ebenso wurden die Effekte auf die Abstinenzraten der Patienten untersucht. Außerdem sollten die Barrieren für die Durchführung von Raucherentwöhnungsmaßnahmen ermittelt werden. Die Studie wurde in einer pneumologischen Akutklinik eines deutschen Universitätsklinikums durchgeführt.

**Methoden:** 24 Ärzte erhielten eine zweistündige Schulung zur Raucherentwöhnung. 109 Kontrollgruppenpatienten, die vor der Schulung in der Klinik behandelt wurden, wurden mit 89 nach der Schulung behandelten Studiengruppenpatienten hinsichtlich der Häufigkeit der ärztlichen Raucherentwöhnungsinterventionen (Ask, Advise, Assist) und ihrer Abstinenzraten drei und sechs Monate nach Klinikaufenthalt verglichen. Die Ärzte schätzten ihre Interventionshäufigkeiten und gaben Gründe an, warum sie in manchen Fällen keine Entwöhnungsinterventionen durchführten.

**Ergebnisse:** Die Anwendung von „Ask“ (OR 3.28, 95% KI 1.13–9.53) durch die Ärzte und die Sechsmonats-Abstinenzraten (OR 2.70, 95% KI 1.24–5.84) waren in der Studiengruppe im multivariablen Modell signifikant höher (p<0.05). Bei „Assist“ zeigte nur das univariable Modell einen signifikanten Einfluss der Schulung (OR 2.05, 95% KI 1.09–3.87). Es wurde kein signifikanter Effekt der Schulung auf die Anwendung von „Advise“ festgestellt. Die Ärzte überschätzten ihre Interventionshäufigkeiten und nannten als Gründe für die Nicht-Durchführung einer Raucherentwöhnung eine wahrgenommene geringe Aufhörmotivation der Patienten und das Vorliegen einer onkologischen oder palliativen Krankheitssituation.

**Fazit:** Eine Ärztekurzschulung zur Raucherentwöhnung in einer pneumologischen Akutklinik erhöht den Einsatz von leitlinienbasierten Raucherentwöhnungsstrategien und könnte einen positiven Effekt auf die Abstinenzraten haben.

**Implikationen:** Die Ergebnisse zeigen, dass Krankenhaus-basierte Strategien wie Ärzteschulungen zur Verbesserung der Raucherentwöhnung nützlich sein können. Strategien zur Überwindung von Hindernissen für die Bereitstellung von Maßnahmen zur Raucherentwöhnung sind erforderlich.

## 1 Introduction

Smoking plays a crucial role in the development of many common diseases. Tobacco is the most common cause of lung cancer and chronic obstructive pulmonary disease (COPD), as well as the leading cause of cancer death worldwide [1], [2]. In Germany, about 110,000 deaths and costs of 79 billion euros are caused by smoking annually [3], [4]. Smoking cessation is a cost-effective and highly powerful method to produce health benefits [5].

Patients with respiratory diseases in particular have an urgent need to stop smoking, since quitting is one of the most important ways to improve their prognosis [6], [7]. These patients may be especially motivated while being hospitalized for a smoking-related disease and while being confronted with respiratory symptoms [8], [9]. Thus, health care providers play an important role in smoking cessation [10], [11], [12]. In routine clinical practice, smoking cessation is essential and should be offered to all patients, especially to those with respiratory diseases [13], [14].

There are evidence-based smoking cessation strategies which can be implemented in busy clinical settings with minimal requirements for providers. The 2008 Clinical Practice Guideline Treating Tobacco Use and Dependence by the US Public Health Service [15] suggests the 5As intervention:

1. ask the patient about tobacco use,
2. advise him or her to quit,
3. assess willingness to make a quit attempt,
4. assist those who are willing to quit,
5. arrange follow-up contact to prevent relapse [15].

In smokers unwilling to quit, the use of motivational interviewing can increase cessation rates significantly [15], [16]. The 5As are an effective short intervention that can be used when time or training requirements do not permit more intensive counseling – which are conditions found in acute care hospitals as in the setting of this study [14], [17], [18], [19]. There is only little data on hospital-based smoking cessation programs in Germany. There is evidence that hospitalized patients are receptive to bedside cessation counseling [13].

Despite the existing effective strategies and the undisputed health benefits, smoking cessation offers are not sufficiently implemented in the German health care system, particularly not in pulmonary care [20], [21], [22]. The number of smokers receiving smoking cessation counseling during contacts with health care services in Germany is low as compared to data from other countries [23], [24]. Only about 240 hospitals and other health care institutions are currently members of the German Network for Tobacco Free Healthcare Services (Deutsches Netz Rauchfreier Krankenhäuser & Gesundheitseinrichtungen, DNRfK e.V.). Only half of them are certified with “silver status”, which means they provide activities in professional qualification and staff training in smoking cessation.

Numerous trials showed that training health professionals in smoking cessation improves the knowledge about and the frequency and quality of performed smoking cessation interventions [25], [26], [27], [28]. A meta-analysis of Carson et al., which included studies with a large range of training intensity, also showed a significant effect of training on abstinence rates [25]. Only few studies have been published that examine the effect of short training programs of less than 2 hours – comparable with the training intensity in this study – on abstinence rates, and these studies show contradictory results [29], [30], [31], [32], [33]. Thus it is still unclear which training intensity is required to influence smoking behavior. To our knowledge, studies examining the effectiveness of short physician training programs in German hospitals and in the special setting of acute-care pulmonology departments have not been conducted yet.

The aim of this study was to explore the effect of a short, two-hour physician training on the physicians’ use of guideline-related smoking cessation strategies in the setting of an acute-care pulmonology department. Another objective was to analyze the effect of the training on the patients’ cessation rates. Furthermore, we aimed to assess the physicians’ attitudes and their perceived barriers for the delivery of smoking cessation interventions.

## 2 Methods

### 2.1 Study design and participants

We conducted a prospective, controlled clinical trial with a pre/post design in the pulmonology department of a German university hospital, the Saarland Medical Center (Homburg, Germany) between 2012 and 2014. 24 physicians received a two-hour training in smoking cessation.

We compared data of 109 control group patients (pre-training group, data collected before the training within a quality control routine survey) to data of 89 intervention group patients (post-training group, data collected with written informed consent after the training).

The study participants were in- and outpatients of the pulmonology department, and were active smokers or recent ex-smokers (defined as having quit less than one year ago) according to their self-declared smoking status. The number of inpatients included in the study was higher than the number of outpatients. This is due to the structure of the department in general, where the number of outpatients is lower than the number of inpatients, and due to the lower readiness of outpatients to participate in the study. 

### 2.2 Assessment instruments

As illustrated in Table 1 (Tab. 1), 650 questionnaires were handed out in the pre-training period and 750 in the post-training period to newly-admitted patients to screen them for their smoking status. Of these, 513 and 469 filled questionnaires were returned, respectively. In these questionnaires, patients declared their smoking status by choosing one of these options: *I am a non-smoker; I am a recent smoker having quit more than one year ago; I am a recent smoker having quit less than one year ago; I am an active smoker*. In addition, they filled out the Hospital Anxiety and Depression Scale (HADS-D) [34] and the Fagerström Test for Cigarette Dependence (FTCD) [35], [36], [37]. Ex-smokers answered the Fagerström Test according to their former smoking behavior.

126 of the patients in the pre-training group and 138 of those in the post-training group met the inclusion criteria. Inclusion criteria were being an active smoker or a recent ex-smoker having quit less than one year ago according to the self-declared smoking status. A biochemical validation of the smoking status was not carried out. 17 vs. 49 of those patients were excluded because they met the exclusion criteria. These were the disability to participate in the discharge interview (T1) due to poor health, or unavailability for the discharge interview (T1). Finally, we included 109 vs. 89 patients who participated in the discharge interview (T1).

The mean length of stay during the study period in general was 7.9 days. In the structured discharge interview (T1), patients were asked for socio-demographic attributes, their smoking history, and whether a physician had delivered a smoking cessation intervention during the stay. In detail, the subgroup of active smokers was asked if a physician had 1) screened them for tobacco use, 2) given the advice to quit, and 3) offered any assistance for quitting. The subgroup of recent ex-smokers was asked the first and a modified third question (“Did a physician offer any assistance for the maintenance of abstinence?”). Some participants could not give an answer to these questions. These missing values were not considered and excluded from the calculation of the 3 As (Table 2 (Tab. 2)). Thus, we screened for three, respectively two of the 5 As [15]. We did not screen for the remaining two As of the model (“assess willingness to quit” and “arrange follow-up”), because we considered the method of a patient interview as more precise for this focused approach, and as sufficient for the evaluation of the effectiveness of the training.

Three (T2) and six (T3) months later, we examined the patients’ smoking status during structured telephone interviews. Abstinence was calculated from point-prevalence defined as smoking stop for more than four weeks. Table 1 (Tab. 1) shows the number of dropped-out participants who could not be interviewed at T2 and T3 due to death, poor general health, or non-availability by telephone. In accordance with the Russell Standard, those participants lost to follow-up were regarded as continuing smokers in the calculation of abstinence rates unless they had died [38]. Participants lost to follow-up due to death were excluded from the calculation. At T2, 9 patients of the pre-training group and 3 of the post-training group were lost to follow-up by death; at T3, there were 8 vs. 4 patients. 11 patients of the pre-training group reentered the hospital between T2 and T3 when the post-training period had already started. Thus they were treated by trained physicians before their six-month follow-up data were collected. They were also considered as continuing smokers in the calculation of six-month abstinence rates to prevent a bias.

Pre- and post-training group were compared with regard to the patient-reported frequencies of the three As performed by the physicians and the three- and six-month abstinence rates.

### 2.3 Survey on the physicians’ attitude towards smoking cessation

To identify reasons for not delivering a smoking cessation intervention, we carried out a survey one year after the training (Attachment 1). In a questionnaire, physicians were requested to estimate the percentages of their performed “Ask, Advise, Assist” interventions to characterize their self-perception. The mean values of these estimations were compared to the patient-reported frequencies in the post-training group. In form of free-text answers, physicians described barriers to the provision of the elements Ask, Advise to quit, and Assist.

### 2.4 Description of the intervention

The physician team consisted of 6 attending physicians and 18 residents during the study period. The physician training comprised two hours of information about the basics of tobacco dependence and the smoking cessation strategy of the 5 As and the 5 Rs (Attachment 2, p. 3) based on the method of motivational interviewing [39] and represented in the Clinical Practice Guideline Treating Tobacco Use and Dependence by the US Public Health Service [15]. At the end of the training session, physicians practiced cessation counseling in a simulation training with actors. A standard operation procedure (SOP) for smoking cessation was put at the physicians’ disposal and implemented at the entire department (Attachment 2 ).

### 2.5 Data analysis

Data analysis was performed using IBM SPSS Statistics Standard 20. In a univariate analysis, Pearson’s chi-square test was used in qualitative scales to compare the outcomes and some characteristics of pre- and post-training group. For the comparison of the remaining patient characteristics, we applied a t-test for normally distributed variables, and a Mann-Whitney U test for not normally distributed interval-scaled and ordinal variables. In a multivariable model, a binary logistic regression in which patient characteristics are included as covariables was used in order to adjust the association between period (pre vs. post as independent variable) and the outcomes (3 As and abstinence rates as dependent variables). Covariables were sex, active smoker or recent ex-smoker, cigarette dependence (FTCD), anxiety (HADS), depression (HADS), level of education, and the presence of lung cancer as categorical variables, as well as age and number of pack years as continuous variables. Due to low case numbers, the level of education was entered into the model as a dichotomous variable, summarized into lower level (lower secondary and secondary school) and higher level (high-school, college degree).

The patient characteristic of „in- and outpatient“ could not be included in the model due to low case number.

## 3 Results

### 3.1 Drop-out analysis and descriptive statistics of pre- and post-training group

We registered the smoking status of 982 patients in both groups, of which 133 (13.5%) were active smokers (64 (12.5%) in the pre-training group and 69 (14.7%) in the intervention group), and 131 (13.3%) were recent ex-smokers (62 (12.1%) in the pre-training group and 69 (14.7%) in the intervention group. 109 of 126 registered active smokers and recent ex-smokers were included into the pre-training group, and 89 of 138 into the post-training group. After three months, 92 (84.4%) of the pre-training group patients and 81 (91.0%) of the post-training group patients could be interviewed on the telephone. After six months, we could follow up with 61 (56.0%) and 73 (83.2%), respectively (Table 1 (Tab. 1)). The causes for dropping out of the follow-up interviews were death (17 (15.6%) in the pre-training group and 8 (9.0%) in the post-training group), poor general health (3 (2.8%) vs. 0), and non-availability by telephone (17 (15.6%) vs. 7 (7.9%)).

In addition, 11 (10%) patients of the pre-training group dropped out at T3 for the reason mentioned above. Table 3 (Tab. 3) compares different patient characteristics of the pre- and post-training group to verify their comparability.

### 3.2 Short training of physicians increases the use of tools for smoking cessation

In order to find out how the short smoking cessation training influences physicians’ use of guideline-related smoking cessation tools, we compared the statements of the pre- and post-training group patients on the physicians’ performances of “Ask, Advise, Assist”. In case of “Advise to quit”, only the subgroup of active smokers was considered.

As illustrated in Table 2 (Tab. 2), the number of patients reporting that a physician had screened them for smoking (“Ask”) and offered assistance (“Assist”) is about three times (“Ask”) and two times (“Assist”) higher in the post-training group (OR(Ask)=3.07, OR(Assist)=2.05) than in the pre-training group. In the multivariable logistic regression, in which patient characteristics are included, the significant effect on “Ask” was confirmed (OR=3.28), but there was no significant effect seen on “Assist”. The rate of patients reporting they had been advised to quit by a physician was 11.4% higher in the post-training group, but this difference was not statistically significant. Hence, it can be stated that physicians who have received smoking cessation training are significantly more likely to perform “Ask” and probably “Assist” than untrained physicians.

The multivariable logistic regression showed the following effects of patient characteristics on the physicians’ use of cessation intervention tools: Physicians tend to use “Ask” and “Advise” more in younger patients than in older patients. Physicians were more likely to use “Assist” in patients without lung cancer, patients with a positive anxiety score, and in active smokers compared to recent ex-smokers.

### 3.3 A short training for physicians results in significantly higher six-month abstinence rates in the overall collective and the subgroup of smokers

We next aimed to measure the impact of the physician training on the patients’ smoking behavior by comparing abstinence rates of pre- and post-training group after three and six months. The physician training had no effect on the three-month abstinence rates. However, the likelihood that patients reported being abstinent after six months in the overall collective (according to the multivariable analysis) and in the subgroup of smokers (according to the univariable analysis) was higher (OR=2.70, OR=3.41 respectively) in the post-training group. The logistic regression models analyzing the abstinence rates of the two subgroups (active smokers and recent ex-smokers) separately were not statistically significant due to low case numbers.

Abstinence rates of recent ex-smokers were not influenced (Table 4 (Tab. 4)). Patient characteristics considered in the multivariable analysis had no significant influence on cessation rates.

The logistic regression models analyzing the abstinence rates of the two subgroups (active smokers and recent ex-smokers) separately were not statistically significant due to low case numbers.

### 3.4 Physicians overestimate themselves in the application of cessation tools as compared to the patient-reported cessation interventions

Physicians were asked to estimate the percentages of patients they usually ask about their smoking status, of smokers they advise to quit, and of active smokers and recent ex-smokers to whom they offer assistance for quitting or for maintaining abstinence. We compared the averaged self-estimated values to the results of the patient interviews of the post-training group.

15 physicians – clinical interns or board-certified pulmonologists – answered the questionnaires. Two thirds of these physicians had attended the smoking cessation training. Physicians tended to overestimate the frequency of implemented smoking cessation interventions, especially regarding the second and the third “A”. Physicians correctly assessed the fact that they did not advise every identified smoker to quit, and that they did not offer assistance to every identified smoker and recent ex-smoker (Table 2 (Tab. 2)).

### 3.5 Reasons for not applying cessation instruments

In the second step of the survey, physicians were asked to give reasons for not performing an “Ask, Advise, Assist” intervention. The results are shown in Table 5 (Tab. 5). The most abundant answers were: forgetting, recognition of the patients’ low motivation and poor compliance, poor health, and an oncological disease including a palliative care situation.

## 4 Discussion

The main finding of the present study was that the short two-hour training of physicians in an acute care pulmonology department leads to an increase in the use of guideline-based cessation strategies (Ask and Assist) by these physicians, and to higher smokers’ cessation rates six months after their discharge. Physicians tended to overestimate themselves in the application of cessation tools. The main barriers physicians reported for not performing a smoking cessation intervention were the perceived patients’ low motivation to stop, an oncological disease including a palliative care situation, and forgetting.

The Clinical Practice Guideline Treating Tobacco Use and Dependence by the US Public Health Service [15] as well as the S3 Guideline on Smoking Cessation by the Association of the Scientific Medical Societies in Germany [12] strongly suggest that smokers should receive a smoking cessation intervention when they come in contact with health professionals. A hospital stay can be considered an instructive moment for changing smoking behavior [40]. Not only should active smokers be helped to stop smoking, but recent ex-smokers should be helped to maintain their abstinence, as the risk of relapse of the latter is high, especially in the early stages of quitting [41]. In the present study, we found that many patients who came to the pulmonology department had recently quit smoking and considered themselves former smokers. The rate of current smokers was only 13.5%. Therefore, former smokers who quit less than a year ago were also included in the study.

The number of completed questionnaires at T0 varied between the pre-training and post-training group (78% vs. 62%). Furthermore, the proportion of registered smokers and recent ex-smokers which were included in the study and interviewed at T1 also varied between both study groups (86% vs. 64%). The reasons for these differences are not entirely clear; the lower availability of study personnel in the post-training group and changes in the seasonal distribution of the patient cohort could have contributed. We assume that patients who were not motivated to participate in smoking cessation were more likely not to participate and that this may have influenced internal validity and caused a selection bias in favor of the intervention.

Although physicians were taught the 5 A approach in the training, we only investigated the use of 3 of the 5 As in the patient interviews: Ask, Advise to quit, and Assist. We did so because these 3 As – screening for tobacco use, giving the advice to quit, and offering assistance – are the essential elements of a short intervention. In addition, we did not consider a structured patient interview as an appropriate instrument to capture the other two As of the model (“Assess willingness to quit” and “Arrange follow-up”), because they are less concise, and difficult for the patient to detect during the physician’s cessation intervention. Patients probably would not have noticed if a physician had evaluated their motivation to quit. “Arrange follow-up” is not clearly distinguishable from “Assist” for the patient, and the clinical setting does not provide a routine structure for arranging a follow-up intervention. Therefore we decided not to assess these two As. We considered this focused approach sufficient for the evaluation of the training’s effectiveness.

In the present study, we examined for the first time the effects of a short physician training in smoking cessation in the setting of a pulmonology university department. The positive influence of training on the frequency of performed smoking cessation interventions shown in numerous studies could be confirmed for a training program in this particular setting [25], [26], [27]. A training of two hours was sufficient to improve the physicians’ performance of Ask, and probably also of Assist. The significant effect on Assist could only be seen in the univariate analysis, not in the multivariable model where outcomes were adjusted to patient characteristics. A reason might be the lower case number in the multivariable analysis due to a higher missing item number in this model. The fact that even this small training effort can be effective is confirmed by findings from recent studies in other health care settings such as in primary care [30], [31], [32], [33]. Physicians tended to use “Ask” and “Advise” more in younger patients than in older ones. Physicians were more likely to use “Assist” in patients without lung cancer, with a positive anxiety score, and in active smokers compared to recent ex-smokers. This partly confirms the results of the physician survey, which showed that physicians tend to differentiate on the basis of certain patient characteristics whether to perform a smoking cessation intervention or not.

A second main finding of our study was that training may have a positive effect on the patients’ cessation rates. The abstinence rates after six months were significantly higher in the post-training group. The abstinence rates after three months were not significantly different, but showed a trend towards higher rates in the post-training group. This result of a higher effect after six months compared to the effect after three months is rather unusual. The different drop-out numbers in the two groups at T2 and T3 might have led to this unusual trend and to an overestimation of the effect of the training on cessation rates in general. As we used the method of a conservative estimate of cessation rates – which means that drop-outs were regarded as continuing smokers (unless they had died) –, and the drop-out rates in the pre-training group were higher than in the post-training group, cessation rates in the pre-training group might have been underestimated and the effect of the intervention overestimated.

However, abstinence of the recent ex-smokers was not improved. The fact that there was more relapse between three and six months in the pre-training group than in the post-training group might show that our intervention had the sustainable effect of stabilizing the achieved abstinence. Meta-analyses have shown that abstinence rates can be increased by training health professionals [25]. However, these calculations included programs with heterogeneous training intensities, with durations ranging from 40 minutes to several days. Four of six studies with comparable training intensities of 2 hours or less did not show any significant effect on abstinence. However, there were low increases in abstinence rates of about 4–5% [31], [32], [33], [42]. The smokers’ six-month abstinence rate in our study increased by about 19.4 % in the post-training group, which was higher than expected.

In contrast to the cited studies, which were predominantly conducted in the primary care system, the present study took place in a hospital department of pulmonology with a tertiary acute health care setting. Half of the participants were diagnosed with lung cancer, one quarter with COPD. The participants were more motivated to stop smoking according to the Stages of Change Model compared to patients in a primary care setting [36]. Being hospitalized for a smoking-related disease such as COPD and lung cancer and suffering from respiratory symptoms might be strong motivators to quit smoking [8], [9], [43], [44], [45]. This might have positively influenced cessation therapy adherence and cessation success. The present study took place in a particular setting with a highly selected sample. It therefore only allows limited conclusions to be drawn for other settings in tertiary care. The recent ex-smokers turned out to be a problematic group whose smoking behavior was not influenced by the intervention. This finding is consistent with a meta-analysis which showed that effective therapy strategies to prevent relapse after quitting are not yet available [46].

A further aim of the present study was to analyze why physicians did not perform smoking cessation interventions in all patients, even after having received training. The physicians’ estimations show that they tend to overestimate the frequency of their interventions as compared to the patient-reported frequencies. This confirms observations of recent studies which showed that intervention frequencies reported by health providers tend to surpass patient-reported frequencies [47], [48]. This distorted self-perception might be a first obstacle for providing an intervention and should be reported back to the physicians. In accordance with the patients’ reports, physicians correctly assess the fact that they do not advise every identified smoker to quit, and that they do not offer assistance to every identified active smoker and recent ex-smoker. When being asked for reasons, the physicians’ answers predominantly referred to the patients’ low motivation to quit, poor compliance, poor health, an oncological disease, and a palliative situation. Lack of skills, which was stated as a central barrier in many physician surveys, was mentioned only twice, which can be seen as a success of the training [49], [50]. The perception of the patients’ low motivation to stop is a commonly reported barrier in health professional surveys [49], [51], [52]. This group of consent smokers seems to discourage physicians to discuss a smoking stop. Trainings should underline the importance of motivating the consent smoker who is unwilling to quit by using the effective techniques of motivational interviewing. Further reasons for not performing smoking cessation referred to patient-related factors: a palliative care situation and reduced life expectancy, or the diagnosis of an oncological disease. Conducting a smoking cessation intervention in the named situations was considered inappropriate by the physicians – a barrier which was not reported in comparable surveys conducted in primary care, a cardiology ward, or an emergency department [51], [52]. In our study setting of a pulmonology tertiary acute care facility, the number of patients that suffered from lung cancer or severe COPD and were in a final stage situation was high. This led to an uncertainty among the physicians whether to discuss a smoking stop or not. In the survey, physicians described that they felt less comfortable to discuss a smoking stop with this group of patients. Guideline recommendations consider smoking cessation an important component of lung cancer therapy. However, it should be discussed for the individual case whether a smoking cessation intervention is appropriate in a palliative care situation or not. Future trainings in comparable settings should focus on this group of patients and provide opportunities to discuss how to treat it.

The present study has some methodological limitations: “Ask” for smoking status was only investigated for active smokers and recent ex-smokers. To focus on the most relevant target group, we decided to only evaluate active smokers and recent ex-smokers, while excluding non-smokers. As we conducted structured interviews to collect these data, it would have meant a significantly greater effort to interview non-smokers as well. The relation between in- and outpatients was unequal in the two compared groups, with a higher number of inpatients in the post-training group. This might have led to a more intensive cessation counseling in the post-training group and an overestimation of the success rates. Smoking status was not verified by a biochemical validation such as carbon monoxide measurement. Application frequencies of the 3 As were based on patient reports and were not examined by objective methods such as video documentation. The study population was too small to conduct all analyses needed. The study should be repeated with a higher case number. Observers who collected the data in structured discharge and follow-up interviews were aware of the study period (pre- vs. post-training). This could have introduced an outcome assessment bias in favor of the intervention. The external validity of the findings is limited since the study was conducted in one clinical department. A multicenter study is urgently needed to reinvestigate the findings.

## 5 Conclusion and practice implications

The present study showed that a short physician training in smoking cessation is an effective measure to improve cessation counseling, and that it may improve abstinence rates in the setting of an acute care pulmonology department. However, even after having received training, physicians did not perform a smoking cessation intervention in every case. Physicians perceived patients with a low motivation to quit, poor compliance, poor health, an oncological disease, or a palliative care situation as problematic groups, which prevented the physicians from performing a smoking cessation intervention.

The findings indicate that short physician training programs in smoking cessation should be implemented to promote hospital-based smoking cessation in pulmonology departments. Future trainings in comparable settings should focus on cessation strategies for the recent ex-smoker and the consent smoker unwilling to quit. Furthermore, they should provide opportunities to discuss the problematic field of smoking cessation in patients with oncological diseases or in palliative care situations. The findings suggest that physicians tend to overestimate their intervention frequencies. Making them aware of this fact within a training program might be a possible starting point to improve their performances.

## Notes

### Ethical statement

Approval for the study was obtained from the Ethics Committee of the Saarland Medical Association (ID 162/12).

### Authors’ contributions

Volker Köllner and Robert Bals contributed equally to this manuscript

### Competing interests

The authors declare that they have no competing interests.

### Acknowledgments

We thank the department of Medical Biometrics, Epidemiology and Medical Information Processing of Saarland University for their excellent consulting in statistical analysis. We are also grateful to Stefanie Mireisz for her support in data collection.

## Supplementary Material

Physician survey form (German)

Standard operating procedure (SOP) for smoking intervention interviews (German)

## Figures and Tables

**Table 1:**
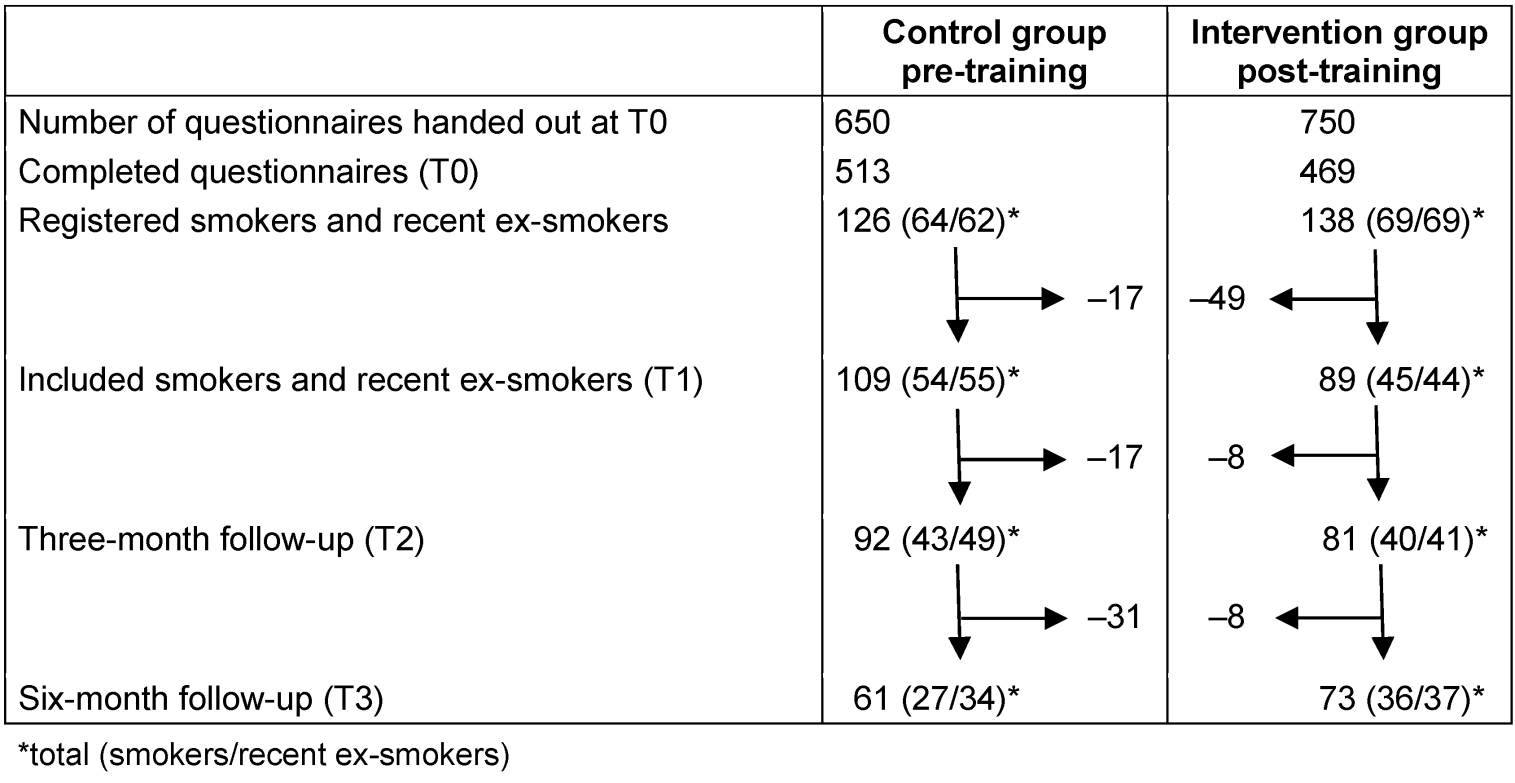
Enrollment and drop-out analysis with the different points of data collection (T0–T3)

**Table 2:**
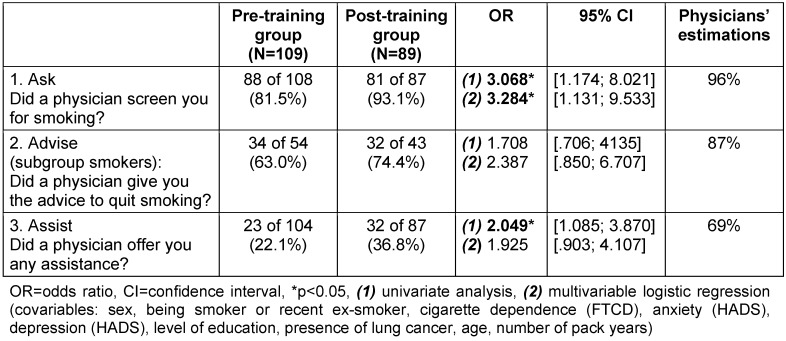
Physicians’ use of smoking cessation tools before and after smoking cessation training and the physicians’ self-estimations

**Table 3:**
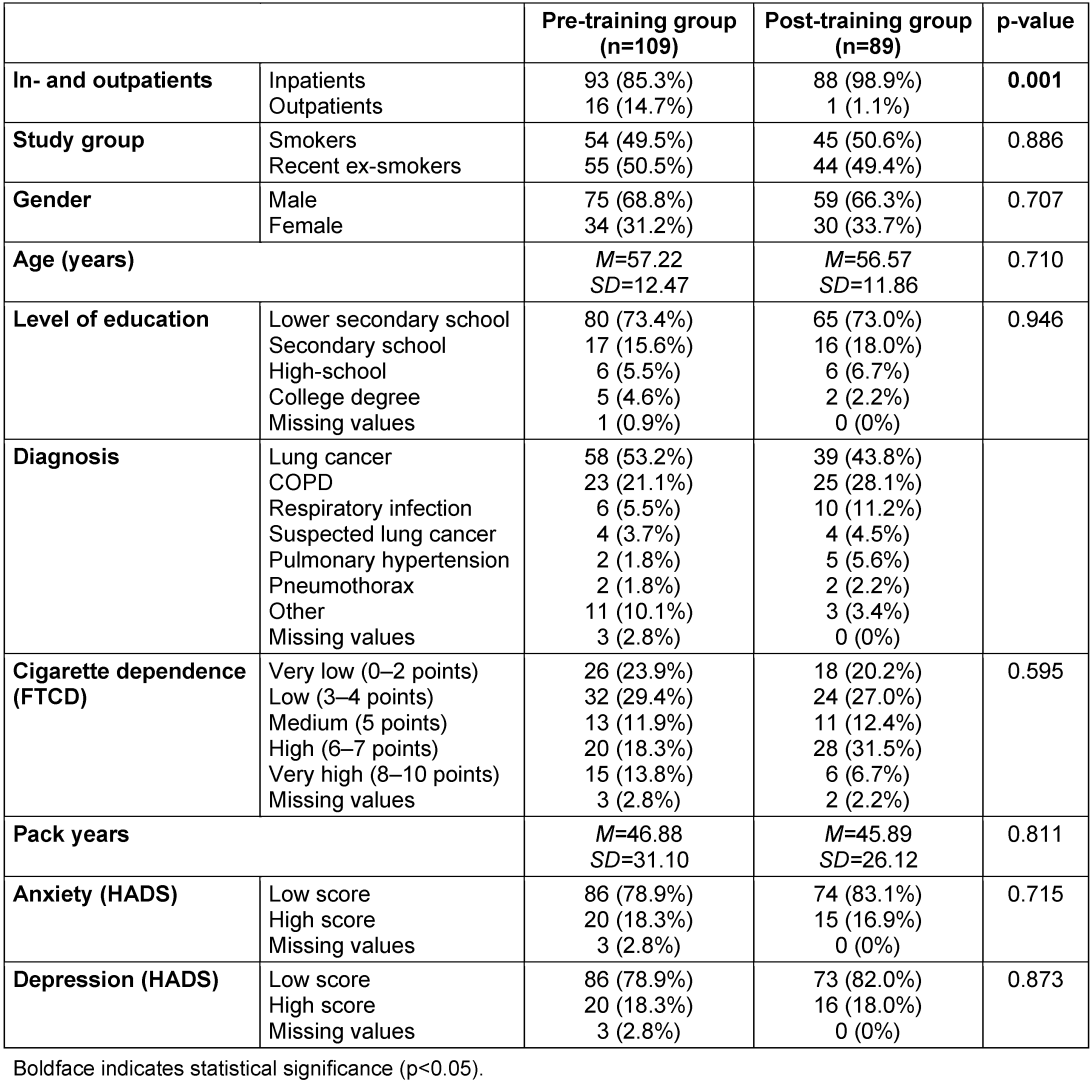
Comparison of pre- and post-training group

**Table 4:**
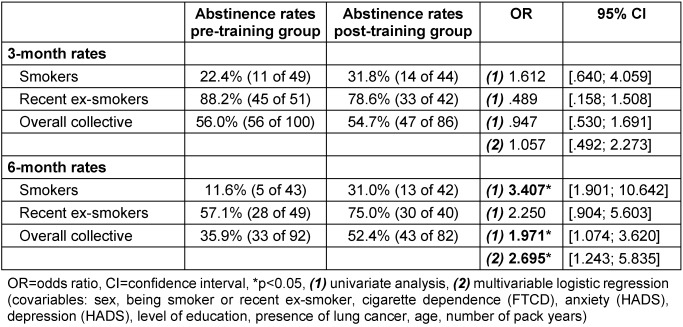
Comparison of pre- and post-training group abstinence rates

**Table 5:**
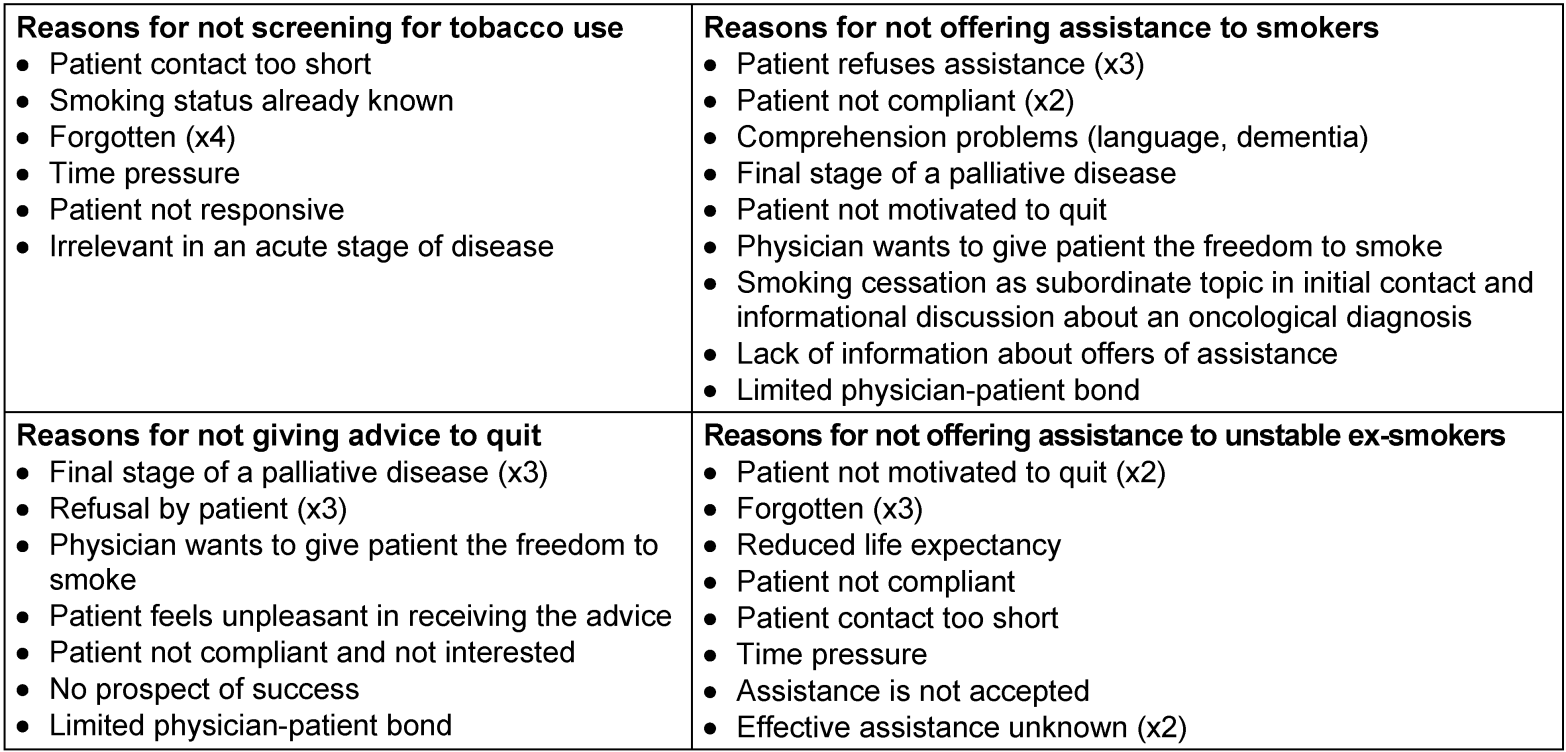
Results of the survey on the physicians’ attitude towards smoking cessation, showing the physicians’ reasons for not applying cessation instruments
