# Supplementary material for: The effects of a short-term physician training on smoking cessation in a university pulmonary department
Source: Ger Med Sci. 2020 Jul 3;18:Doc06. doi: 10.3205/000282 (PMC7373096; doi:10.3205/000282)
Supplement: Physician survey form (German) [file GMS-18-06-s-001.pdf]

Attachment 1: Physician survey form (German)

Attachment 1 to:

Bauer A, Brenner L, Moser J, Trudzinski F, Köllner V, Bals R. The effects of short-term physician training on smoking cessation in a university pulmonary department. GMS Ger Med Sci. 2020;18:Doc06. DOI: 10.3205/000282, URN: urn:nbn:de:0183-0002826

Ärztebefragung

-im Rahmen der Raucherentwöhnungsstudie-

Sehr geehrte Ärztinnen und Ärzte,

dies ist eine Befragung im Rahmen der Raucherentwöhnungsstudie. Es wäre sehr nett, wenn Sie den folgenden Fragebogen ausfüllen könnten. Der ausgefüllte Bogen kann per Mail an Prof. Bals gesendet oder im Chefsekretariat abgegeben werden.

Vielen Dank!

□ männlich □ weiblich

□ Assistenzarzt/-ärztin □ Facharzt/-ärztin □ Oberarzt/-ärztin

Haben Sie an der internen Schulung zu Raucherentwöhnung im Oktober 2012 teilgenommen?

□ Ja □ Nein

Wie schätzen Sie sich selbst hinsichtlich der folgenden Fragen ein?

1. - In wie viel Prozent fragen Sie die Patienten nach ihrem **Rauchstatus**?

   - Wenn nicht in 100%:
     Können Sie Gründe nennen, warum Sie nicht immer nach dem Rauchstatus fragen?

2. - In wie viel Prozent **raten** Sie rauchenden Patienten **mit dem Rauchen aufzuhören**?

- Wenn nicht in 100%:
  Können Sie Gründe nennen, warum Sie nicht immer den Rat erteilen mit dem Rauchen aufzuhören?

3. - In wie viel Prozent bieten Sie Rauchern eine **Hilfestellung zur Entwöhnung** an?

- Wenn nicht in 100%:
  Können Sie Gründe nennen, warum Sie nicht jedem Raucher Hilfe anbieten?

4. - In wie viel Prozent bieten Sie instabilen Exrauchern (aufgehört innerhalb des letzten Jahres) **Hilfestellung zur Aufrechterhaltung** an?

- Wenn nicht in 100%:
  Können Sie Gründe nennen, warum Sie nicht jedem instabilen Exraucher Hilfe anbieten?
