# Supplementary material for: The effects of a short-term physician training on smoking cessation in a university pulmonary department
Source: Ger Med Sci. 2020 Jul 3;18:Doc06. doi: 10.3205/000282 (PMC7373096; doi:10.3205/000282)

Attachment 2: Standard operating procedure (SOP) for smoking intervention interviews (German)

Attachment 2 to:

Bauer A, Brenner L, Moser J, Trudzinski F, Köllner V, Bals R. The effects of short-term physician training on smoking cessation in a university pulmonary department. GMS Ger Med Sci. 2020;18:Doc06. DOI: 10.3205/000282, URN: urn:nbn:de:0183-0002826

Klinik für Innere Medizin V

SOP

Raucherentwöhnung **Version vom 10.12.2012**

Diese SOP beschreibt die Vorgehensweise zur Gesprächsführung zur Entwöhnung von Rauchern.

Zur Einschätzung der Abhängigkeit vom Rauchen kann der FTND angewendet werden:

<u>FAGERSTRÖM-Test zur Beurteilung der Nikotinabhängigkeit (FTND)</u>

- Wann rauchen Sie nach dem Aufstehen die erste Zigarette?
  - Innerhalb von 5 Minuten — 3 Punkte
  - Innerhalb von 6–30 Minuten — 2 Punkte
  - Innerhalb von 31–60 Minuten — 1 Punkt
  - Nach mehr als 60 Minuten — 0 Punkte

- Finden Sie es schwierig, an Orten, an denen das Rauchen verboten ist, nicht zu rauchen?
  - Ja — 1 Punkt
  - Nein — 0 Punkte

- Auf welche Zigarette würden Sie nicht verzichten wollen?
  - Die erste am Morgen — 1 Punkt
  - Andere — 0 Punkte

- Wie viele Zigaretten rauchen Sie pro Tag?
  - Mehr als 30 — 3 Punkte
  - 21–30 — 2 Punkte
  - 11–20 — 1 Punkt
  - 10 oder weniger — 0 Punkte

- Rauchen Sie in den ersten Stunden nach dem Erwachen im Allgemeinen mehr als am Rest des Tages?
  - ja — 1 Punkt
  - nein — 0 Punkte

- Kommt es vor, dass Sie rauchen, wenn Sie krank sind und tagsüber im Bett bleiben müssen?
  - ja — 1 Punkt
  - nein — 0 Punkte

Beurteilung der Abhängigkeit: 0–2 Punkte: keine/sehr gering, 3–4 Punkte: gering, 5 Punkte: mittel, 6–7 Punkte: stark, 8–10 Punkte: sehr stark

Therapeutische Maßnahmen

- **Keine/sehr geringe Abhängigkeit:** FTND: 0–2, <10 Zigaretten/Tag
  - Raucherberatung, Kurzintervention, evtl. Nikotinkaugummi (2 mg)

- **Mittelschwere Abhängigkeit:** FTND: 3–4, <20 Zigaretten/Tag
  - Raucherberatung, Kurzintervention, Gruppenbehandlung, Nikotinpflaster alternativ Nikotinkaugummi (4 mg)

- **Schwere Abhängigkeit:** FTND : 5–6, 20–30 Zigaretten/Tag
  - Raucherberatung, Kurzintervention, Gruppenbehandlung, Nikotinpflaster alternativ Nikotinkaugummi (4 mg), bei starken Entzugserscheinungen Kombinationstherapie aus Pflaster und Kaugummi, evtl. längerdauernde Substitutionstherapie

- **Sehr schwere Abhängigkeit:** FTND: >6, >30 Zigaretten/Tag
  - Raucherberatung, Kurzintervention, Einzel- oder Gruppenbehandlung, Nikotinpflaster in Verbindung mit Nikotinkaugummi (4 mg), alternativ Nasalspray, evtl. längerdauernde Substitutionstherapie

CAVE: Kontraindikationen der Nikotinersatztherapie beachten!

Motivierende Gesprächsführung (MI):

Empathie ausdrücken, Widerstand aufnehmen, Diskrepanz entwickeln, Selbstwirksamkeit fördern

Die fünf „A“ als Leitfaden für die Inhalte des Gesprächs

ASK: Abfragen des Rauchstatus

ADVISE: Anraten des Rauchverzichts

ASSESS: Ansprechen der Aufhörmotivation. Es soll geklärt werden, ob der Raucher bei diesem Kontakt bereit ist, einen Termin für einen Rauchstopp zu vereinbaren. Falls keine Bereitschaft besteht → **5 „R“**

ASSIST: Assistieren beim Rauchverzicht. Aktive Unterstützung bei dem Rauchstoppversuch anbieten

ARRANGE: Arrangieren der Nachbetreuung (Rückfallprophylaxe)

Die fünf „R“: Strategien zur Überzeugung zum Rauchverzicht

RELEVANCE: Relevanz des Rauchverzichts aufzeigen (körperl./familiäre/soziale Bedenken)

RISKS: Risiken des Rauchens benennen

REWARDS: Reize und Vorteile des Rauchstopps verdeutlichen

ROADBLOCKS: Hindernisse und Schwierigkeiten des Rauchstopps ansprechen

REPETITION: Erneutes Ansprechen bei jedem Folgekontakt

Weitere Infos/Ansprechpartner:

- **Rauchertelefon: 01805-313131**
- **www.bzga.de**
- **www.rauch-frei.info**
- **www.dkfz.de**

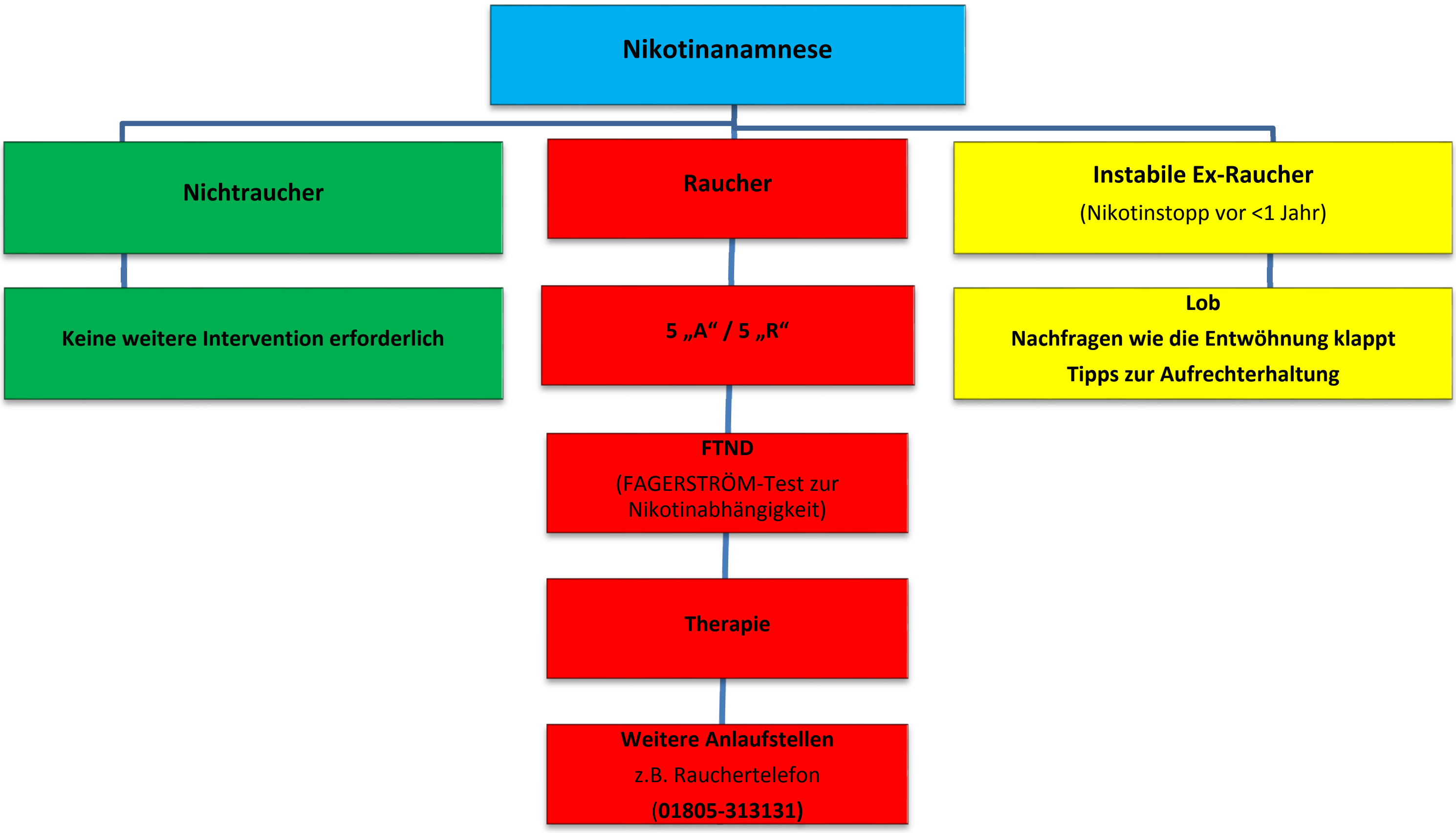

Supplement: Standard operating procedure (SOP) for smoking intervention interviews (German) [file GMS-18-06-s-002.pdf]
